# Gemcitabine-loaded poly (lactide-co-glycolide) – poly (ethylene glycol) gold nanoparticles: Evaluating the effect of fabricating methods on physicochemical characteristics, in vitro drug release and cytotoxicity

**DOI:** 10.1007/s13346-025-01884-y

**Published:** 2025-06-04

**Authors:** Adibah Shakri, Khairunnisa Mohd Paad, Norazalina Saad, Izzat Fahimuddin Mohamed Suffian

**Affiliations:** 1 Department of Chemical and Environmental Engineering, Malaysia-Japan International Institute of Technology (MJIIT), Universiti Teknologi Malaysia Kuala Lumpur, 54100 Jalan Sultan Yahya Petra, Kuala Lumpur Malaysia; 2 Department of Applied Science, Muroran Institute of Technology, Hokkaido, 050-8585 Japan; 3 UPM-MAKNA Cancer Research Laboratory (CANRES), Institute of Bioscience, Universiti Putra Malaysia, 43400 Serdang, Selangor, Malaysia; 4 Department of Pharmaceutical Technology, Kulliyyah of Pharmacy, International Islamic University of Malaysia, Jalan Sultan Ahmad Shah, Bandar Indera Mahkota, 25200 Kuantan, Pahang, Malaysia

**Keywords:** Electrospray, MTT assay, Drug release kinetics, Gemcitabine, PLGA-PEG

## Abstract

Hybrid gold nanoparticles (AuNPs) are defined as a combination of organic and inorganic materials used as nanocarriers in the drug delivery system to enhance the performance of the drugs. Methods to fabricate the hybrid AuNPs such as double emulsion and nanoprecipitation require several steps and are inconsistent in their physicochemical characteristics limiting their application in drug delivery. The electrospray method has become a powerful tool for fabricating nanoparticles due to its simplicity, rapidity and consistency. Hence, this study aims to evaluate the performance of hybrid AuNPs prepared by two different methods; double emulsion and electrospray including the physicochemical characteristics, drug release profile and in vitro cytotoxicity. Hybrid AuNPs were prepared using double emulsion and electrospray methods. Then, the formed particles' hydrodynamic size, polydispersity index (PDI) and zeta potential were characterised using a Zetasizer Nano ZS. Furthermore, the drug release kinetics and cytotoxicity effects were evaluated using the dialysis method and the MTT assay. Hybrid AuNPs formed by the electrospray method (HAES) exhibit smaller particle sizes at 147.00 ± 10.4 nm with a PDI value of 0.35 ± 0.01 and zeta potential of -31.5 ± 2.6 compared to hybrid AuNPs formed using double emulsion methods (HADE) which exhibit larger particle sizes of 287.47 ± 1.87 nm with PDI value of 0.25 ± 0.09 and zeta potential of -23.30 ± 0.6. This study suggests that HAES prevent burst effects during drug release due to polymer composition changes under different temperatures that can enhance the drug delivery system. From the MTT assay, at 50 mg mL^−1^, the HAES exert higher toxicity effects of 50% compared to HADE due to different physicochemical characteristics and drug release patterns. These findings emphasise the advantage of the electrospray method in fabricating polymeric gold hybrid nanoparticles by forming smaller particle sizes, stable formulation, and higher toxicity to cancer cells compared to particle synthesis by the double emulsion method.

**Graphical Abstract:**

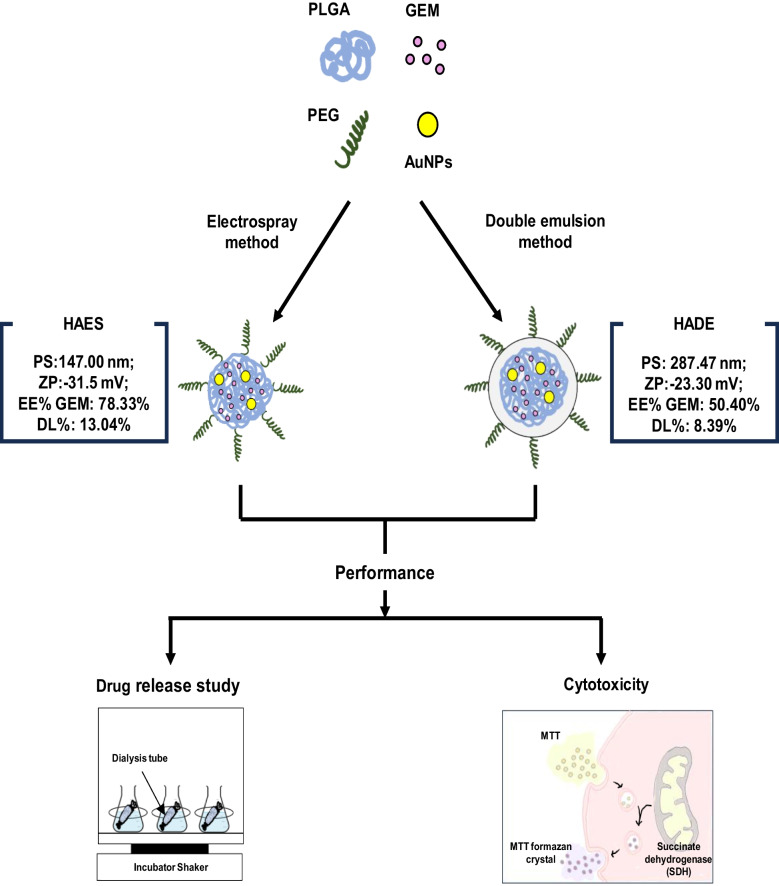

## Introduction

Gemcitabine (2’, 2’ difluoro 2’-deoxycytidine, dFdC), is a cytidine analogue and is known as a broad-spectrum anticancer drug that is being used for the treatment of pancreas, colon, breast, bladder and ovarian cancers [1]. It is a cell-cycle specific (S-phase) cytotoxic agent that kills cells undergoing DNA synthesis [2]. Currently, gemcitabine (GEM) is used as a standard cervical cancer treatment in Malaysia and is administered in its analogue form [3]. However, this hydrophilic drug delivery poses challenges such as limited biodistribution [4], potential side effects [5] and drug resistance [6]. To overcome this limitation, nanocarriers such as polymer-based nanoparticles, lipid-based nanoparticles and metal-based nanoparticles have been developed to enhance drug delivery. The use of PLGA-PEG polymer nanoparticles successfully encapsulates this hydrophilic drug and improves therapeutic efficacy by sustaining the drug release and increasing the bioavailability of GEM. This results significantly enhance the cytotoxicity of GEM (IC50 17.59 ± 2.91 ng mL^−1^) compared to GEM nanoparticles (7.62 ± 0.84 ng mL^−1^) in Panc1 cell line [7]. Single material formulation has been shown to improve drug delivery through sustained drug release, increase bioavailability at the target and efficacy. Here, gold nanoparticles (AuNPs) are known for their unique characteristics of surface plasmon resonance (SPR) that uses light to generate heat that can be used for thermal therapy in cancer cell treatment [8]. Because of the advantages possessed by the gold nanoparticle, a hybrid nanoparticle formulation was proposed in this study. Hybrid nanoparticles is a combination of more than two materials that exhibit a dual function that enhances their properties to better serve as a drug delivery system.

Several methods such as nanoprecipitation, emulsification and solvent displacement have been used for synthesising hybrid nanoparticles [9]. Double emulsion has been extensively used for the past 30 years due to its ability to encapsulate hydrophobic and hydrophilic molecules [10]. The double emulsion also known as water-in-oil-in-water (W/O/W) emulsion, involves dispersion of the aqueous phase (water) in the organic phase (oil), followed by re-emulsification in another aqueous phase [11]. This technique enables the encapsulation of both hydrophilic and hydrophobic molecules, as the aqueous phase dissolved the hydrophilic molecules while the organic phase solubilised the hydrophobic molecules. Moreover, since double emulsion consists of both phases, it facilitates the co-encapsulation of both hydrophobic and hydrophilic in the same system [12]. However, this method requires several steps of first emulsion, second emulsion and solvent evaporation which is time-consuming and the possibility of sample loss during the process [13]. Furthermore, each step requires different solvents and the by-products cannot be easily removed and may induce toxicity to the biological system [14] Besides that, this method also involves high-energy processes of mixing and stirring which can influence the stability of the formulation resulting in broad particle size distribution which is very crucial in drug delivery [15].

In contrast, the electrospray method incapacitates its main drawbacks and limitations. Electrospray has a one-step direct method making it efficient, cost-effective and reducing the potential loss of samples [16]. In addition, the one-step method of electrospray ensures complete drying of the solvent used due to high voltage supplies to the nozzle that formed the Taylor-cone jet mode leaving only the final product minimising the intoxication potential [17]. Furthermore, electrospray consists of several controllable parameters such as nozzle size that can control the size distribution [18]. In designing nanosystems, particle size is the most crucial for improving drug delivery and targeting [19]. Different synthesis methods produce different particle characteristics responsible for drug delivery and targeting.

Therefore, to overcome the limitation of double emulsion, we aim to evaluate the physicochemical characteristics, in vitro drug release and cytotoxicity of the electrospray method as an alternative method in fabricating hybrid nanoparticles. In the present work, the polymeric gold hybrid nanoparticle (PEGylated PLGA, AuNPs and GEM) was fabricated using the double emulsion and one-step electrospray methods. Then, the physicochemical characteristics, encapsulation efficiency, drug release behaviour and cytotoxicity of the hybrid AuNPs formed by the electrospray method (HAES) and hybrid AuNPs formed using double emulsion methods (HADE) were evaluated to determine the size, particle stability, encapsulation capacity, prolonged drug release and the ability to kill cancer cells. This study highlights the advantages of electrospray in producing monodispersed and stable formulations while showing a prolonged ‘no-burst’ effect of the drug release, potentially improving the drug delivery system and the therapeutic outcomes.

## Materials and methods

### Materials

Poly (D-lactide-co-glycolide), (PLGA, MW ~ 40 000–75 000 g mol ^−1^, P2066, Sigma Aldrich, Dorset, UK) and poly (ethylene glycol) (PEG, MW: 3400 g mol ^−1^, P9906, Sigma Aldrich, Dorset, UK) were conjugated to form PLGA-PEG as described in our previous work [20]. Then, the conjugated PLGA-PEG was used in the encapsulation process to form PLGA-PEG hybrid gold nanoparticles (AuNPs). Gold nanoparticles (spherical, 50 nm, 44.5 µg mL^−1^, Cytodiagnostics, Burlington, Canada). The size validation has been conducted using DLS and UV–vis (supp. material). Gemcitabine (GEM) (MW 263.20 g mol ^−1^, 99.39%, 95,058–81-4, Alfa Aesar, Massachusetts, USA), Tween 80 (0.2%, 98%, Merck, Darmstadt, Germany) was freshly prepared before each use. Acetone (99%, R&M Chemicals, Selangor, Malaysia), phosphate-buffered saline (1X, PBS, pH 7.4, Thermo Fisher Scientific, USA) and dimethyl sulfoxide (DMSO, 99.9%, Thermo Fisher Scientific, USA) were used as received without any further treatment otherwise stated. The HeLa (HPV18), a cervical cancer cell used as a cancer cell line, was kindly supplied by the Institute of Bioscience at Universiti Putra Malaysia. HeLa cells were cultured in Dulbecco’s modified Eagle medium (DMEM) with 10% fetal bovine serum (FBS) and 5% penicillin–streptomycin (Thermo Fisher Scientific, USA) at 37 ℃ in a humidified atmosphere containing 5% CO_2_. 3-(−4,5-dimethyl-2-thiazolyl)−2, 5-diphenyl-2H-tetrazolium bromide (MTT) powder (98%, M2128, Sigma Aldrich, Dorset, UK).

### Preparation of hybrid gold nanoparticles (AuNPs) using double emulsion method

**Fig. 1:**
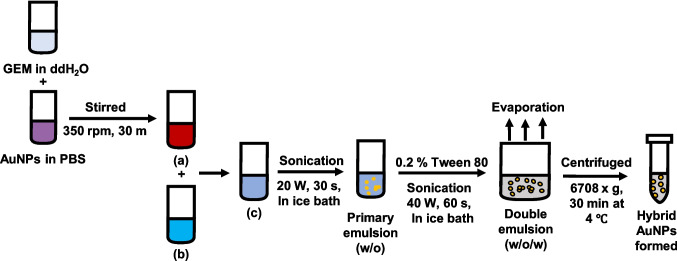
Illustration of hybrid AuNPs preparation using double emulsion (HADE). GEM in ddH_2_O was mixed into gold in PBS forming solution **(a)** (aqueous phase) and then mixed into solution **(b)** (PLGA-PEG in acetone-organic phase) becoming solution **(c).** Then, solution **(c)** was sonicated and became the primary emulsion (w/o). Then, Tween 80 was added and sonicated again to obtain a second emulsion. The water–oil-water emulsion was then centrifuged and HADE formed were dispersed in Tween 80 and stored at 4 ℃. Abbreviation: GEM-Gemcitabine, ddH_2_O-double distilled water, AuNPs-gold nanoparticles, w-water, o-oil

Hybrid gold nanoparticles (AuNPs) were prepared using the double emulsion solvent evaporation method as illustrated in Fig. 1 [21]. Briefly, 5 mg mL^−1^ GEM was prepared in distilled water (aqueous solution). 1 mL of 2.5 µg mL^−1^ AuNPs solution was added dropwise to the aqueous solution and stirred for 30 min at 350 rpm forming solution (a). Then, 25 mg PLGA-PEG was dissolved in 5 mL acetone (organic solution) (solution b) and was added to the aqueous solution forming solution c. Solution c was then emulsified for 30 s at 20 amp in an ice bath using an ultrasonicator (QSonica 700, 20 kHz, 700 W, QSonica LLC, USA) forming a primary emulsion (water-in-oil). For the second emulsion, 20 mL of 0.2% Tween 80 was added and emulsified again for another 1 min at 40 amp, in an ice bath. The final formed double emulsion was stirred overnight at room temperature at 350 rpm under a fume hood to facilitate the complete evaporation of acetone. Next, the form hybrid particles were centrifuged (MiniSpin 5452, Eppendorf, Germany) in 2 mL centrifuge tubes at 6708 × g for 30 min at 4 ℃. Finally, the hybrid AuNPs formed using double emulsion methods (HADE) were dispersed in Tween 80 for storage at 4 ℃ prior use.

### Preparation of hybrid gold nanoparticles for electrospraying

Hybrid gold nanoparticles (AuNPs) were prepared via the electrospray method. Briefly, AuNPs in PBS were added to PLGA-PEG (dissolved in acetone at 25 mg in 5 mL^−1^) and stirred (MS-MP4, Daihan Scientific, Toa Payoh, Singapore) at 350 rpm for 30 min at room temperature to allow complete dissolution into Mixture A (AuNPs and PLGA-PEG mixture). Then, GEM was dissolved in deionised water before being added to Mixture A to form an electrospraying formulation. The mixture was then stirred at 350 rpm for 15 min at room temperature.

**Fig. 2:**
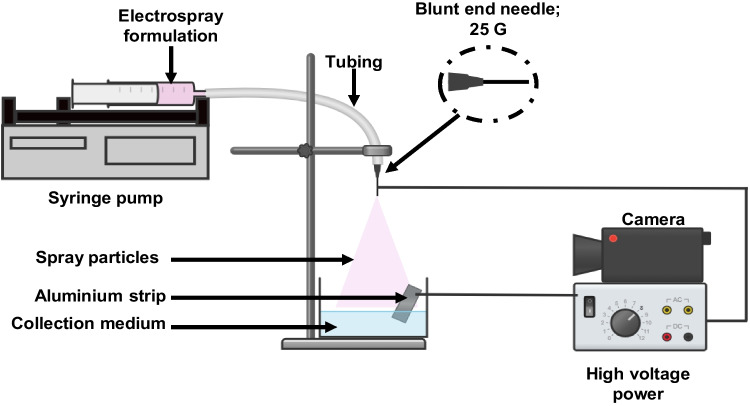
Schematic diagram of the electrospray setup used. The electrospray setup consists of high-voltage power, a digital camera, a collector, a syringe pump and a syringe with a blunt-end needle (25G). Additionally, fixed parameters were applied including an electrospraying formulation of 5 mL, a tubing length of 18 cm (connecting the syringe and the needle), and a collection distance of 5 cm (from needle tip to petri dish) was applied

Then, 5 mL of prepared electrospray formulation was subjected to an electrospray setup as illustrated in Fig. 2 [22]. The electrospray setup resides in a closed transparent chamber made of acrylic with a size of 200 cm × 100 cm to minimise external influence. Inside the chamber, a high-voltage power supply (PS35-PV, Nanolab Instruments, Malaysia) connects the 25G blunt end needle to the aluminium strip immersed in a collection medium (20 mL of 0.2% Tween 80). A high-performance digital camera (X4, Shenzen Haiweixun Electronics Co. Ltd, China) was equipped in the chamber to ensure a Taylor cone jet mode throughout the experiment. The centre of the setup consists of a syringe with the 25G blunt end needle (Terumo Europe N.V, Belgium), automated syringe pump (NSL-20, Nanolab Instrument, Malaysia), tubing (TYGOPRENE™ TYGON XL-60, AN800007, Darwin Microfluids, France) and a glass petri dish as a particle collector (100 mm × 15 mm able to hold approximately 20 mL liquid). A constant condition of the electrospray setup was established; 10.7 kV power supply with a flow rate of 0.5 mL h^−1^, a tubing length of 18 cm (connecting the syringe and the needle) and a collection distance of 5 cm (from needle tip to petri dish). The electrosprayed nanoparticles were collected into a glass petri dish and were kept in a 15 mL centrifuge tube at 4 °C for physicochemical characteristics and encapsulation efficiency analysis. The electrospray formulation was injected through the syringe using the syringe pump into the electric field to form the hybrid nanoparticles. The solution formed spherical droplets of hybrid AuNPs formed by the electrospray method (HAES) and was collected in a glass petri dish using a collection medium and was kept at 4 ℃.

### Measurements of particle size, zeta potential, and size distribution of HAES and HADE

Dynamic light scattering (DLS) measurements were conducted to determine the hydrodynamic diameter (Z-average) and the size distribution of hybrid AuNPs with a particle size analyser (Nano-ZS90, Malvern Instruments Co. Ltd, UK) at a scattering angle of 90°. The size distribution was analysed based on intensity-weighted measurement, and the hydrodynamic diameter (Z-average) was reported. The HAES and HADE samples were dispersed in deionised water at a dilution factor of 1:1000. 1 mL of the prepared sample was added to the disposable polystyrene cuvette and the small bubbles in the solution were removed before measurement to ensure uniform dispersion of particles. Measurements were performed at room temperature (25 ℃) with an equilibrium time of 120 s. Each sample was measured in triplicate, and the results were expressed as mean ± standard error (SE). The polydispersity index (PDI) was also recorded for size distribution. The zeta potential was also measured on folded capillary cells and the values were calculated by the instrument using the Smoluchowski equation [23].

### Encapsulation efficiency

**Fig. 3:**
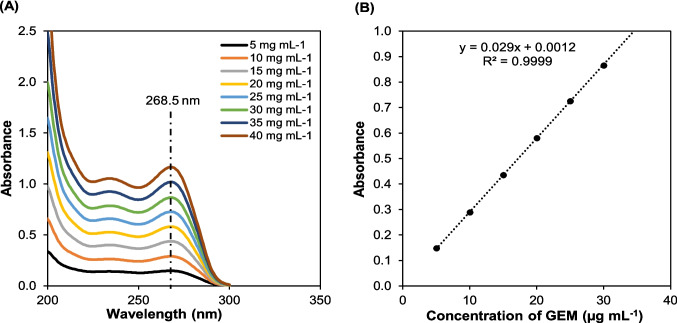
Absorbance profile of GEM using UV–vis spectroscopy. **(A)** UV–vis absorption spectra for different concentrations of GEM in distilled water at RT (25 ℃) and **(B)** standard linear calibration curve of GEM over different concentration ranges from 5- 40 mg mL^−1^. GEM was dissolved in distilled water at several concentrations ranging from 5–40 mg mL^−1^ and measured using UV–vis spectroscopy. Results showed the highest absorbance peak at 268.5 nm and the standard curve plotted with a high R^2^ value of 0.9999 indicated that the data fit the calibration curve and can be used for GEM estimation in this study. Abbreviation: GEM-gemcitabine, RT-room temperature

**Fig. 4:**
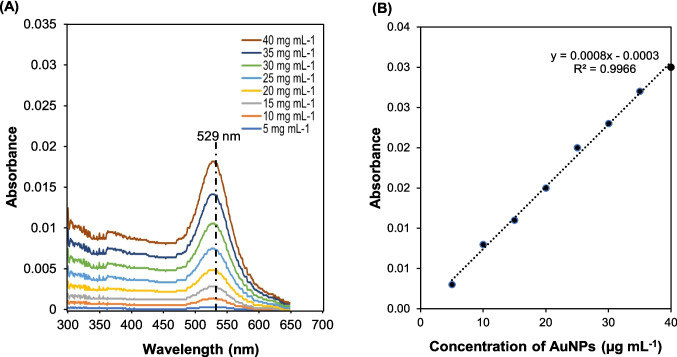
Absorbance profile of AuNPs using UV–vis spectroscopy.** (A)** UV–vis absorption spectra for different concentrations of AuNPs in distilled water at RT (25 ℃) and **(B)** standard linear calibration curve of AuNPs over different concentration ranges from 5–40 mg mL^−1^. AuNPs were dissolved in distilled water at several concentrations ranging from 5–40 mg mL^−1^ and measured using UV–vis spectroscopy. Results showed the highest absorbance peak at 529 nm and the standard curve plotted with a high R^2^ value of 0.9966 indicated that the data fit the calibration curve and can be used for AuNPs estimation in this study. Abbreviation: AuNPs-gold nanoparticles, RT-room temperature

The encapsulation efficiency (EE%) measurement of the HAES and HADE was performed on a dual–beam UV–Vis Spectrophotometer (UV-1800, Shimadzu, Japan) at the wavelength of 268.5 nm, standard OD for GEM [24] and 529 nm, standard optical density (OD) for gold nanoparticles (AuNPs) [25] in a quartz cuvette of 3 cm path length. Briefly, 1 mL of HAES and HADE was centrifuged for 30 min at 10,000 rpm and 4 ℃ temperature. Then, 150 µL of the supernatant for each formulation was dissolved in 3 mL of deionised water. The measurement was conducted on the supernatant assuming all encapsulated HAES and HADE were collected in the pellet. Quantification of GEM and AuNPs was estimated from a calibration curve of concentration-dependent in a concentration range of 5–40 mg mL^−1^ of each GEM (Fig. 3A and 3B) and AuNPs (Fig. 4A and 4B). The encapsulation efficiency, EE (%) and drug loading, DL (%) were calculated using Eq. 1 and Eq. 2 [18] as follows:

1 $$EE\left(\%\right)=\frac{Encapsulated\ drug\ or\ AuNPs\left(Drug/AuNPs-drug/AuNPs\ free\right)}{Initial\ amount\ used\ in\ the\ nanoparticles}\times 100$$

2 $$DL\left(\%\right)=\frac{Total\ drug-free\ drug}{Total\ mass\ of\ nanoparticles}\times 100\%$$

### In vitro drug release profile and kinetic behaviour

The in vitro release profile of the GEM from HAES and HADE was conducted using a dialysis membrane method (68100, 10 kDa, Thermo Fisher, Massachusetts, US) [26]. The dialysis membrane was soaked in distilled water overnight before the experiments to smooth the membrane. Briefly, 75 mL phosphate-buffered solution of pH 7.4 and 5 mL of 0.1% Tween 80 were prepared as release media in a 150 mL glass beaker. The initial free-GEM concentration is 1.25 mg mL^−1^ and HAES and HADE were prepared to achieve a 1.25 mg mL^−1^ GEM concentration equivalence of the formulation as the initial concentration (final volume of 3 mL). Then, the formulation was placed into the dialysis bag, submerged in release media using a burette stand and placed on the magnetic stirrer (37 °C, 150 rpm). At predetermined time intervals (0, 2, 4, 8, 12, 24, 48, 72 and 96 h), 3 mL of drug release media was aspirated and replaced with 3 mL fresh release media to maintain constant bath volume. Drug concentration was determined by measuring the absorbance of GEM at 268.5 nm using a UV–vis spectrophotometer (Fig. 3A) and calculated using a calibration curve (Fig. 3B). The measurements were done in triplicate (*n* = 3).

The obtained data was also analysed with several mathematical models to determine the best kinetic model to investigate the release of GEM from the hybrid formulations by fitting it into the zero-order, first-order, Higuchi, Hickson Crowell, and Korsmeyer-Pepas models, respectively [27]. The mathematical models are shown as follows;

Zero-order release kinetic model;

3 $$Q={Q}_{0}+Kt$$

First-order release kinetic model;

4 $$\frac{Q}{{Q}_{0}}=1-{e}^{-Kt}$$

Higuchi release kinetic model;

5 $$Q=K{t}^{0.5}$$

Hixon-Crowell release kinetic model;

6 $${Q}^\frac{1}{3}- {Q}_{0}^\frac{1}{3}= Kt$$

Korsmeyer – Pepas release kinetic model;

7 $$\frac{Q}{{Q}_{0}}=K{t}^{n}$$

where *Q* represents the amount of drug released at the time, *t,* ${Q}_{0}$ is the initial amount of dru*g, K* is the release constant and n represents the release exponent.

### In vitro cytotoxicity evaluation

The cytotoxicity of HAES and HADE was determined using an MTT assay on HeLa cells (P12, HPV18, cervical cancer cells) [28]. Briefly, HeLa cells were seeded at 1 × 10^4^ cells per well using a 96-well plate (Corning, Fisher Scientific, U.K) and incubated at 37 ℃ with 5% CO_2_ overnight. The cells were treated with the HAES and HADE with different GEM concentrations equivalent (encapsulated drug) ranging from 1.56–100 µg mL^−1^ for 72 h at 37 ℃. Desirable GEM concentrations were prepared by serial dilution from a stock solution because the final formulation of hybrid AuNPs is dispersed in a surfactant. The nanoparticle mass used to achieve the concentration was calculated based on DL%. For HADE, the nanoparticle mass needed to achieve the GEM concentrations is between 0.018 mg mL^−1^–1.19 mg mL^−1^. For HAES, the nanoparticle mass needed to achieve the GEM concentrations is between 0.01 mg mL^−1^–0.76 mg mL^−1.^ The empty PLGA-PEG used as a control is 25 mg mL^−1^. The untreated cells were used as a negative control. After that, the media was removed and washed using PBS. 100 µL serum-free media with 20 µL of MTT (5 mg mL^−1^) reagent was added to each well plate and incubated for 4 h, allowing viable cells to reduce MTT to purple-coloured formazon crystal. Subsequently, 200 µL DMSO was added to each well to dissolve the formazan crystal. Cell viability was measured using a microplate reader (Infinite 200 Pro, Tecan, Switzerland) at 570 nm with the results presented as a value of mean ± SE. The experiment was conducted in triplicate and the cell viability was determined using Eq. 8 [29] as follows:

8 $$Cell\ viability\left(\%\right)=\frac{Optical\ density\ of\ treated\ cells}{Optical\ density\ of\ untreated\ cells}$$

### Statistical analysis

All numerical data reported are expressed as mean ± standard error (SE) of the number of replicates *n* = 3. Statistical analysis was calculated using GraphPad Prism® (Version 8; GraphPad Software, San Diego, USA). Multiple t-tests were conducted to compare means between different synthesis methods at a 5% confidence level to determine whether a significant difference exists in the average mean between groups. The *ρ*-values ≤ 0.05 were considered statistically significant. The significance level is indicated as *ρ < 0.05, **ρ < 0.01 and***ρ < 0.001.

## Results and discussion

This study focused on fabricating hybrid AuNPs to identify the most effective method for enhancing the efficacy of the drug delivery system. Hybrid AuNPs designed for passive targeting were synthesised using two fabrication methods; the double emulsion method and the electrospray method. Key physicochemical characteristics of the synthesised nanoparticles were evaluated, and their performance was assessed through in vitro drug release and cytotoxicity studies, as reported below.

### Fabrication method's effects on physicochemical characteristics of the HAES and HADE

Dynamic light scattering (DLS) was utilised to quantify the physicochemical properties of HAES and HADE using a Zetasizer Nano ZS, and the results are summarised in Table 1. DLS analysis shows a Z-average hydrodynamic diameter of 287.47 ± 1.87 nm, which is significantly larger with a 73.82% increase than that of empty PLGA-PEG (75.26 ± 1.35 nm) for HADE indicating that the integration of AuNPs and GEM into PLGA-PEG forming HADE. Similarly, for HAES, a Z-average hydrodynamic diameter of 147.00 ± 10.4 nm was observed with a significant increase of 36.21% from empty PLGA-PEG (93.77 ± 1.48 nm). It is revealed that HAES exhibit 48.86% smaller particle sizes, 147.00 ± 10.4 nm than HADE, 287.47 ± 1.87 nm. Similarly, Xiong et al. has also reported that polymer and hydrophilic chemotherapy formed particle sizes of 225 nm when prepared using double emulsion methods [30]. In addition, several other authors reported that particles fabricated via electrospray methods formed particle sizes between 140–200 nm [18, 29]. The size difference might be attributed to the process involved in both techniques where the double emulsion principle relies on sheer force and the mechanical agitation of the homogenisers, creating a wide-size dispersion of droplets [10, 11]. Furthermore, the electrospray setup primarily includes a nozzle that controls the particle diameter, alongside other key factors such as the flow rate, polymer concentration and nozzle distance to the particle collector [31]. Particle size distribution is the key characteristic in designing a nanocarrier to improve drug delivery. This study's nanoparticle design focuses on passive targeting through the EPR effect. The EPR effect facilitates and increases drug accumulation within tumours by exploiting the unique characteristics of tumour vasculature [32]. To take advantage of the distinctive characteristics of the tumour vasculature, particle sizes should ideally be between 10–300 nm to avoid kidney filtration and elimination by macrophages [33]. In this study, both methods produced particles within a size range of ≤ 300 nm, indicating their suitability for generating nanoparticles ideal for drug delivery applications. However, other research found that particles smaller than 200 nm cause more obvious effects as they improve circulation, biodistribution and cellular uptake into cancer cells [34, 35].

**Table 1:** Droplet size, polydispersity index (PDI) and zeta potential of empty PLGA-PEG formulation, HADE and HAES. Results are expressed as mean ± SE (*n = 3*). Statistical analysis was performed using multiple t-tests. Abbreviation: HADE-Hybrid AuNPs Double Emulsion, HAES-Hybrid AuNPs Electrospray

Nanoparticles	Hydrodynamic diameter (nm)	Polydispersity Index (PDI)	Zeta potential (mV)
Empty PLGA-PEG (DE)	75.26 ± 1.35	0.13 ± 0.02	−10.70 ± 0.3
HADE	287.47 ± 1.87**	0.25 ± 0.09**	−23.30 ± 0.2***
Empty PLGA-PEG (ES)	93.77 ± 1.48	0.33 ± 0.00	−18.45 ± 0.1
HAES	147.00 ± 10.4**	0.35 ± 0.01	−31.50 ± 2.6**

Significance is indicated as *ρ < 0.05, **ρ < 0.01, ***ρ < 0.001

Then, the polydispersity index (PDI) was measured and showed that HADE has significantly 32.43% smaller values at 0.25 ± 0.01 compared to HAES, 0.35 ± 0.01 indicating both methods displayed a monodispersed particle distribution. The polydispersity index (PDI) is used to assess the particle size distribution, with values above 0.4 indicating a broad distribution and values below 0.1 signifying highly uniform, monodispersed particles [36]. Both HAES and HADE exhibited PDI values between 0.25 and 0.35, suggesting low dispersity within the formulation, likely due to aggregation of AuNPs during the synthesis process [37].

Compared to empty PLGA-PEG, the zeta potential of HAES increased by 41.43%, from −18.45 mV to −31.50 mV while the zeta potential of HADE increased by 54.10% from −10.70 mV to −23.30 mV suggesting an interaction between PLGA-PEG, AuNPs and GEM. In addition, a 42% higher zeta potential (−18.45 mV) was exhibited by empty PLGA-PEG of ES compared to empty PLGA-PEG of DE (−10.70 mV). In addition, the same pattern was observed in HAES and HADE, where a 26% higher zeta potential of −31.50 mV in HAES than of −23.30 mV in HADE signifying the influence of different methods on the zeta potential values. In addition, a 26% higher value of −31.5 ± 2.6 mV than HADE at −23.60 ± 0.6 mV was observed indicating HAES demonstrate better formulation stability compared to HADE. Zeta potential allows estimation of colloidal stability where a higher zeta potential indicates long-term colloidal stability of a formulation [38], reflecting a strong interaction between the polymer, gold and drug components. In this study, HAES exhibited a higher particle charge (−31.5 ± 2.6 mV) compared to HADE (23.30 ± 0.2 mV). Similarly, findings from Chatterjee et al. also reported that electrosprayed nanoparticles obtained −36.6 mV and −37.80 mV [29]. The increase in zeta potential is attributed to highly charged droplets formed by the high voltage supplied to the needle. During electrospray, the liquid formulation passes through the needle becomes a highly charged droplet, and breaks into Taylor-cone jet mode to produce a highly charged droplet [31]. In addition, the negative charge may be due to the carboxylic groups (~ COOH) of acid-terminated PLGA available on the surface of nanoparticles [29]. A minimum of zeta potential values of ± 30 mV is necessary to maintain stable dispersion, while values around ± 20 mV indicate short-term stability [38]. Stable formulation (−60 to −100 mV) leads to a stable dispersion of nanoparticles in an aqueous solution through strong electrostatic repulsion with water molecules, which also can prevent aggregation [39].

Overall, HAES exhibited a smaller-size particle (147.00 ± 10.4 nm), a comparable PDI value (0.35) and a higher zeta potential of −31.5 ± 2.6 mV compared to HADE (287.47 ± 1.87 nm, 0.25, −23.30 mV) due to the interaction among PLGA-PEG, AuNPs and GEM forming a hybrid particle via the electrospray method providing long-term stability to the formulation and also suitable for intravenous delivery of GEM for tumour therapy [34].

### Encapsulation efficiency

Encapsulation efficiencies of GEM and AuNPs by the HADE and HAES were tabulated in Table 2. To determine the EE%, the absorbance profiling of both GEM and AuNPs was established using UV–vis spectroscopy and plotted in Fig. 3 (GEM) and Fig. 4 (AuNPs). The EE% drug encapsulated by HAES (78.33 ± 6.70) was significantly higher by 35.66% than HADE (50.40% ± 1.50). Interestingly, the EE% gold encapsulated by HAES (71.00% ± 5.00) and HADE (63.57% ± 6.70) is comparable with only a 10% difference. Higher drug encapsulation by electrospray may be attributed to the technique itself; electrospray forms nanoparticles in an air medium between the needle and collector medium, where it allows for rapid solvent evaporation, leading to the formation of solid nanoparticles with minimal loss of the encapsulated drug. In contrast, even though the double emulsion method is appropriate for hydrophilic drugs, using a liquid medium can facilitate the diffusion of the hydrophilic drug (eg. GEM) to the aqueous phase resulting in lower encapsulation efficiency [29]. Similarly, Hasanpour et al. and Chatterjee et al. also found EE% of 83.3% ± 3.2 and 97.22% exhibited by nanoparticle synthesis via electrospray method [18, 29] that is significantly higher in comparison to nanoparticles prepared by double emulsion (54.89% and 69.01%) [30, 40]. Several strategies could be used to increase the EE% including the use of the co-axial electrospray method, altering the optimum ratio of each component and adding an appropriate surfactant to the formulation [41–44].

**Table 2:** Encapsulation efficiency (EE %) of HADE and HAES. Results are expressed as mean ± SE (*n* = 3). Statistical analysis was performed using multiple t-tests. Abbreviation: HADE-Hybrid AuNPs Double Emulsion, HAES-Hybrid AuNPs Electrospray

Parameters	EE% Drug	DL% Drug	EE% Gold
HADE	50.40 ± 1.50	8.39 ± 0.25	63.57 ± 0.99
HAES	78.33 ± 6.70**	13.04 ± 1.92**	71.00 ± 5.00*

Significance is indicated as *ρ < 0.05, **ρ < 0.01

Drug loading is a crucial parameter in determining the active ingredient encapsulated compared to the total weight of nanoparticles. Additionally, the drug loading (DL%) was calculated and showed a DL% of HAES (13.04% ± 1.92) and HADE (8.39% ± 0.25). In this study, the DL% of HAES and HADE exhibited a higher loading of 13.04% ± 1.92 and 8.39% ± 0.25, respectively, than 7.9% by Saneja et al. and 3.8% by Hamzian et al. where gemcitabine was loaded with PLGA [7, 45]. This suggests that the formulation and composition used in this study may enhance the DL%. One of the factors contributing to this difference is the ratio of drug mass to nanoparticle mass. Saneja et al. achieved 7.9% DL by incorporating a 0.37:1 drug-to-nanoparticle mass ratio. In contrast, a 0.17:1 drug-to-nanoparticle mass ratio was used in this study to achieve 5.8% higher DL% in HADE and 39.41% higher DL% in HAES. This suggests that lower drug to nanoparticles resulted in higher DL% due to the ability of the polymer to encapsulate the drug at a certain amount, exceeding the polymer's capacity to encapsulate the drug will result in lower drug loading due to drug diffusion out from the polymer [46].

Then, comparing two fabrication approaches, HAES (13.04% ± 1.92) exhibited a significant increase of 36.34% in DL% than HADE (8.39% ± 0.25), indicating differences in drug entrapment between the two techniques. Electrospray involves one-step methods to reduce the potential of drug loss resulting in higher entrapment [17]. Double emulsion consists of several steps including solvent evaporation where GEM can diffuse out throughout the process, leading to lower drug loading [13]. Liu et al. found that the drug loading of most nanomedicine is typically low resulting in low clinical translation. Enhanced DL% can reduce the number of nanoparticles required to achieve an optimum therapeutic effect also the systemic toxicity and production cost can be reduced [47].

### Drug release profiles and kinetic behaviour of HAES and HADE

Gemcitabine (GEM) release profiles from HAES and HADE were investigated using UV spectroscopy. GEM standard curves were shown in Fig. 3 indicating a best absorbance at 268.5 nm. To be able to use UV spectroscopy, the GEM standard curve was established using different concentrations of GEM from 5–40 mg mL^−1^ with an R-value of 0.9999 (Fig. 3). The in vitro release of GEM from HAES and HADE was studied in PBS (pH 7.4) at room temperature (25 ℃) and 37 ℃ to mimic the physiological environment.

**Fig. 5:**
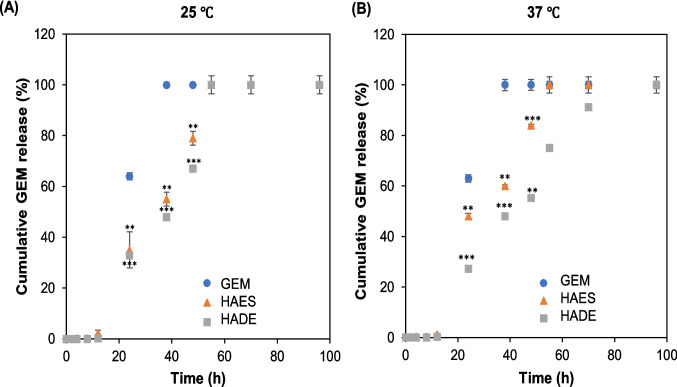
In vitro release profile of free-GEM and GEM from HAES and HADE (PBS, pH 7.4). **(A)** The released profile of free-GEM, GEM from HAES and HADE at 25 ℃. **(B)** The released profile of free-GEM, GEM from HAES and HADE at 37 ℃. Free-GEM showed no significant difference in the GEM released when exposed to different temperatures. At 37 ℃, GEM release from HADE showed a significantly slower and sustained release than that of HAES. In addition, the GEM release from HAES and HADE is significantly slower than that of free drugs. Results are expressed as mean ± SE (*n* = 3). Statistical analysis was performed using multiple t-tests. Significance is indicated as ***p < 0.001, **p < 0.01 and *p < 0.05. Abbreviation: GEM-gemcitabine, HADE-Hybrid AuNPs Double Emulsion, HAES-Hybrid AuNPs Electrospray, PBS-phosphate buffer saline

Figure 5 illustrates GEM's in vitro release profile as a free drug, GEM from HAES and GEM from HADE. Free-GEM shows a biphasic trend with a burst release of 64% before 24 h and sustained release up to 100% after 24 h to 38 h. Similarly, Zhao and Qi also exhibited a biphasic release pattern of free-GEM and a longer release of 70% at 8 h and 100% at 72 h [48]. Furthermore, Lui et al. also exhibited a free-GEM complete release in 24 h [40]. In contrast, Devi et al. and Guarin-Gonzales et al. demonstrate a rapid burst release of free–GEM (100% release within 2–5 h) [6, 49]. The main factor contributing to these discrepancies is the type of GEM used. The studies with a longer release profile (8–72 h) used GEM in its original form without any modification (analogue) while studies that showed rapid release (within 2–5 h) used GEM in its salt form (GEM HCl). GEM (analogue) molecular formula is C_9_H_11_F_2_N_3_O_4_ with a molecular weight of 263.2 mg mol^−1^. Throughout the years, various modifications have been done to GEM, such as chemical modification in the 4-(N)- and 5’- (N) positions of GEM to improve its metabolic stability and cytotoxicity activity [50]. One of the strategies is converting a drug to its salt form to enhance solubility and bioavailability [51]. Gemcitabine hydrochloride (salt form) (GEM HCl) comprises C_9_H_11_F_2_N_3_O_4._HCl, where the HCl functional group is attached to the amine group (N4) of gemcitabine. This addition to GEM HCl enhances its solubility in water. The increased aqueous solubility of the hydrochloride salt is primarily due to its ionic nature, which facilitates better interaction with polar solvents like water [52]. Solubility of GEM (analogue) in water is 0.824 mg mL^−1^ while GEM HCl (salt form) is 58.82 g mL^−1^. This shows that GEM HCl has a significantly higher solubility, approximately 70-fold higher compared to GEM (analogue) in water [53]. Increased solubility of the GEM HCl in water enables faster diffusion, leading to rapid release, and the burst effect appears. In contrast, GEM (analogue) has lower solubility resulting in slower diffusion and release rates. Originally, GEM HCl was utilised for clinical setting purposes specifically for chemotherapy with improved solubility facilitating intravenous administration [50]. However, GEM HCl has been characterised with rapid release and short half-life and may result in systemic toxicity due to frequent dosing to achieve optimal effect [49, 50].

In this study, we aim to formulate a drug delivery system that can improve GEM (analogue) bioavailability and therapeutic performance. GEM (analogue) used in this study is preferable due to its lower solubility compared to GEM (salt form). Higher solubility of GEM HCl may reduce the encapsulation process due to the drug that easily diffuses out from its carrier [54]. Furthermore, GEM in its analogue form is more stable for formulation, especially in aqueous solutions since drugs in their salt form exist in ionised form that are very sensitive to pH. Small changes in the aqueous system will compromise the drug solubility and stability [55]. In addition, GEM in its original form is preferred over its salt form owing to salt form are prone to experiencing hygroscopicity and hydration that will affect their storage, formulation and stability [55].

Free drugs show no significant difference with increased temperature within the first 24 h and reached 100% release in 38 h. In contrast, GEM from HAES and HADE have significantly different release patterns under different temperatures. Regardless of temperature difference, the free-GEM exhibits significantly faster release (63–64% in 24 h) compared to the GEM released from HAES (35–48%) and HADE (27–33%) indicating the use of hybrid AuNPs significantly lowers the release of GEM. For free GEM, it can be observed that the temperature difference does not significantly affect the release of GEM due to its solubility in water. GEM is a hydrophilic drug highly soluble in water [1]. High-solubility drugs readily dissolve in aqueous solutions, leading to rapid and complete release when exposed to aqueous conditions, regardless of temperature [56].

The biphasic trend release (≤ 24 h ≥) was observed for both HADE and HAES where a burst release before 24 h and a sustained release after 24 h. Then, the drug release continued until 100% with HAES for up to 55 h and HADE at 72 h. Notably, HADE released GEM at a slower rate than HAES. Usage of HAES and HADE comprising the PLGA in the formulation has improved the release of GEM by 27% and up to 48% achieving the goal of nanocarrier to maintain and control the drug released over the cancer cells or target sites. Several other researchers noted the same result, demonstrating that PLGA improved the controlled release of bioactive compounds [29, 57, 58]. A study by Wang et al. demonstrated a 95% release in 1 h of free-GEM chloride when compared to PLGA-GEM which achieved 95% within 11 h [59].

**Fig. 6:**
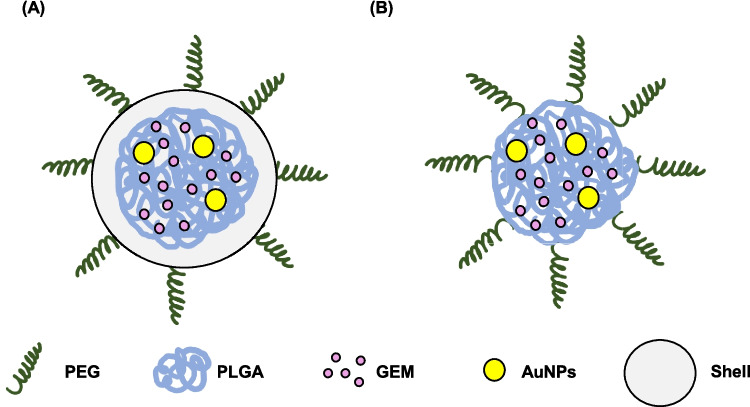
Schematic diagram of possible formation of hybrid AuNPs. **(A)** Possible core–shell formation of HADE **(B)** Possible formation of HAES. This schematic diagram was deduced according to several publications related to this study with similar constituents (polymer PLGA/PEG, drugs) and characteristics (hydrophilic drug). Several studies showed the core–shell formation due to the water and organic phase in the double emulsion [42, 57, 59, 61]. In electrospray, the components are embedded in the polymer matrices [29, 41]. Abbreviation: PLGA-poly-lactide-glycolide-acid, PEG-poly ethylene glycol, AuNPs-gold nanoparticles, GEM-gemcitabine, HADE-Hybrid AuNPs Double Emulsion, HAES-Hybrid AuNPs Electrospray, W/O/W-water in oil in water. The schematic diagram is used to assist in the possible formation of nanoparticles and does not represent the actual data for physicochemical characteristics

Then, the release trend between the HAES and HADE was evaluated resulting in a biphasic trend displayed by both methods. PLGA-related nanoparticles often exhibit biphasic behaviour due to non-encapsulated drugs affixed to the surface anticipating a burst/rapid release. Then, following the drug diffusion from the polymer matrix degradation occurred in a controlled manner due to the molecular weight of the PLGA [60]. This trend was also observed in this study as a rapid release of GEM occurred in the first 24 h, followed by a sustained release after 24 h until the GEM release achieved 100%. To understand the release behaviour, a schematic depiction of the potential formation and entrapment of the GEM and AuNPs was inferred from previous research. Figure 6 shows the possible core–shell structure due to the water and organic phase in the double emulsion [42, 57, 59, 61] while in electrospray, the components are embedded in the polymer matrices [29, 41]. In this study, HAES showed a 23.61% faster release than HADE likely due to the difference in distinct particle nanostructure formation. As depicted in Fig. 6, the core–shell structure formation of HADE formed a shell on the outer layer, thus making drug diffusion more controlled [59].

**Fig. 7:**
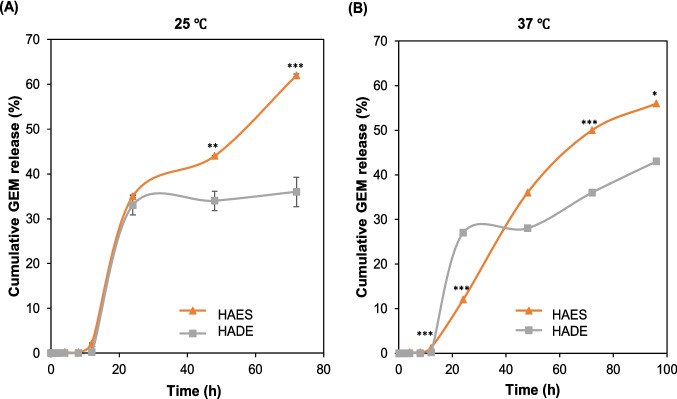
In vitro GEM release profiles between HAES and HADE (PBS, pH 7.4). **(A)** The released profile of GEM from HAES and HADE at 25 ℃. **(B)** The released profile of GEM from HAES and HADE at 37 ℃. At 25 ℃, the GEM release from HAES and HADE showed two types of patterns; before 24 h (burst release) and after 24 h (sustained release). At 37 ℃, the GEM release from HAES showed no burst release indicating a better-controlled release. In addition, the GEM release from HAES is significantly higher than HADE but both have sustained release up to 96 h. Results are expressed as mean ± SE (*n* = 3). Statistical analysis was performed using multiple t-tests. Significance is indicated as ***p < 0.001, **p < 0.01 and *p < 0.05 Abbreviation: GEM-gemcitabine, HADE-Hybrid AuNPs Double Emulsion, HAES-Hybrid AuNPs Electrospray, PBS-phosphate buffer saline

Figure 5 was plotted to see the pattern at 100% released (free-GEM and GEM from hybrid AuNPs). However, to understand more about the biphasic trend, the in vitro GEM profile from HAES and HADE was plotted as shown in Fig. 7. At 25 ℃, the GEM release from HAES and HADE can be categorised into two stages; with a rapid release in the first 24 h, followed by a more controlled and slower release regardless of preparation methods. Surprisingly, at 37 ℃, a different trend of no burst released was exhibited by HAES before 24 h compared to other formulations indicating that temperature affects the GEM release from HAES. A similar finding was demonstrated by Chatterjee et al. that PLGA-Thioguanine synthesised by electrospray showed a sustained release from 60–65% to ~ 95% throughout 60 days of experiments with no indication of a burst effect [29]. On the contrary, the PLGA-Thioguanine synthesised by double emulsion showed a burst release on Day 4 throughout the study. This can be explained by the fact that the hydrolysis of any bond in the matrices has slowed down due to PLGA forming a dense coiled structure when exposed to an electric field during electrospray. Furthermore, a study by Wu et al. combines a neutron reflection (NR) and scattering length density (SLD) to understand the drug/polymer/medium interaction. From here, it stated that when the temperature increases to 37 ℃, the SLD increases, and the water uptake increases to 37.4% increasing the thickness of the polymer layer to 4.31% resulting in a change in the structural composition of the polymer. These changes control the transportation of drugs through the polymer matrix in which the thermal treatment (37 ℃) increases the thickness of the polymer and influences the hydration equilibrium hence the medium molecules are evenly distributed among “molecular pores “of the polymer matrix [62]. Findings from this study suggest that electrosprayed particles under different temperatures contribute to polymer composition changes that prevent burst effects during drug release. This finding shows the importance of electrospray in synthesising temperature-sensitive particles that can enhance the drug delivery system.

Then, the release profile data of the HAES and HADE were analysed by fitting it into five mathematical models, including zero-order, first-order, Higuchi, Hixson-Crowell, and Korsmeyer-Peppas to understand the drug release mechanism. A zero-order model describes the drug release from the system at a constant rate and is only a function of time regardless of concentrations[60]. Meanwhile, the first-order model relates to releasing the remaining hydrophilic drugs within the carrier [63]. Conversely, the Hixson-Crowell cube root model is generally applied to delivery systems whose surface changes over time. The Higuchi model is the most widely used model that characterises drug release from the delivery system through a diffusion mechanism based on Fick’s law [63]. Further analysis of a semi-emperical kinetic approach (Korsmeyer-Peppas) can be utilised to elucidate the specific type of diffusion mechanism by analysing the release exponent “n”. The n value for a spherical system indicates the drug release mechanism; n < 0.43 corresponds to the Fickian diffusion, 0.43 < n < 0.85 indicates the non-Fickian model, and n > 0.85 indicates case II release [64].

**Fig. 8:**
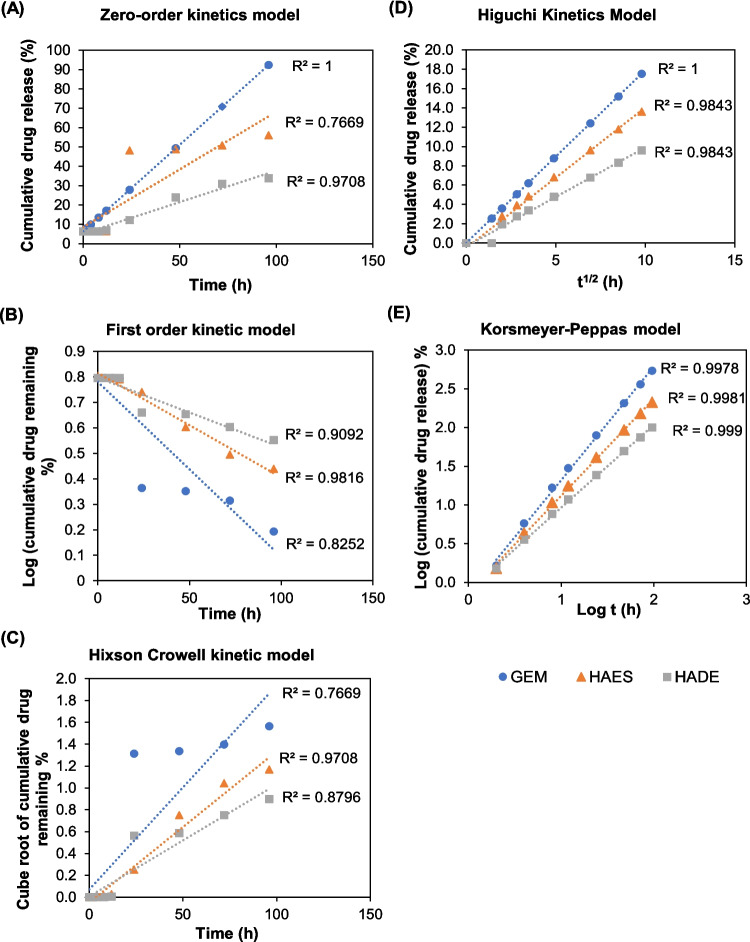
Kinetics profiles from free GEM, GEM from HADE and GEM HAES by fitting to five mathematical models. **(A) **zero-order, **(B)** first-order, **(C)** Hixson-Crowell, **(D)** Higuchi and **(E)** Korsmeyer Peppas kinetics model. The highest R^2^ value for HAES and HADE is the Higuchi model indicating the controlled release mechanism. In addition, the Korsmeyer-Peppas model corresponds to the Fickian diffusion, (n>0.85) indicates the case II release describes the cumulative effect of diffusion and polymer chain relaxation mechanisms. Results are expressed as mean ± SE (*n* = 3). Abbreviation: GEM-gemcitabine, HADE-Hybrid AuNPs Double Emulsion, HAES-Hybrid AuNPs Electrospray

Fitted data of the kinetic modelling and the correlation coefficient values for all applied models were summarised in Fig. 8. The statistical parameter of the correlation coefficient (R^2^) represents the degree of fitting of the model. Both HAES and HADE yielded the highest correlation coefficient (R^2^) of 0.9843 fitted to the Higuchi model, indicating a drug release occurs by diffusion within the delivery system, consistent with the PLGA release mechanism [63]. It is the preferential release mechanism for all loaded nanofibres. Hence, the Korsmeyer-Peppas model was employed to understand the type of diffusion mechanisms. Based on the Korsmeyer Peppas plot, R^2^ value for HAES and HADE is 0.9981 and 0.999 exhibiting a good linearity with release exponent (n) of 1.25 (HAES) and 1.07 (HADE) (> 0.85), indicating a super case II transport mechanism. The super case II transport describes a drug release mechanism characterised by diffusion and polymer chain relaxation or swelling [65]. Several drug release mechanisms from polymeric nanocarriers include diffusion, solvent and degradation-controlled release [66]. The results showed that the drug release mechanism is not solely diffusion-controlled, but is significantly influenced by the erosion mechanism, consistent with the biodegradable nature of polymer nanoparticles. The diffusion mechanism is related to different concentration gradients and once the drug is dissolved inside the core part, it will diffuse from the membrane. A matrix-type system also has a diffusion mechanism in which the drug molecules are evenly distributed in the matrix without a membrane as a barrier resulting in higher release [67]. Similarly, a schematic diagram of electrosprayed hybrid nanoparticles as deduced in Fig. 6 showed no membrane in the system facilitated the diffusion thus explaining a higher release of drug from HAES compared to HADE.

### Cytotoxicity analysis of HAES and HADE

Cytotoxicity analysis of the HAES and HADE was conducted using an MTT assay on cervical cancer cell lines (HeLa cells). MTT assays determine cytotoxicity by measuring the metabolic activity that reflects the estimation of viable cells [28]. MTT assay was an established method and previous research had demonstrated consistent and reliable results when evaluating the toxicity of nanoparticles loaded with GEM in a wide variety of cancer cell lines such as cervical cancer cell lines (HeLa) [68] and non-small cell lung cancer (A549 and H1299) [49]. Moreover, the MTT assay has also been used to determine cytotoxicity from another anti-cancer drug (doxorubicin and cisplatin)[69] and active compound (diferuloylmethane)[70].

HeLa cell is a cervical cancer cell line derived from a human origin established in culture and have been widely used in biological research that offer relevant information to cellular responses [71]. Gemcitabine has a potent effect on several cancer cell lines including cervical cancer cells (HeLa) and the use of HeLa cells suits the purpose of this study [72]. HeLa cell is an epithelial cell in the cervix (adenocarcinoma) that might limit the information on cytotoxicity studies. Further study may use other cervical cancer cell lines such as CaSki, SiHa, ME-180 and C33 A that cover broader classifications such as squamous cell carcinoma and adenosquamous carcinoma [73]. This limitation will not affect the result of the cytotoxicity in this study.

**Fig. 9:**
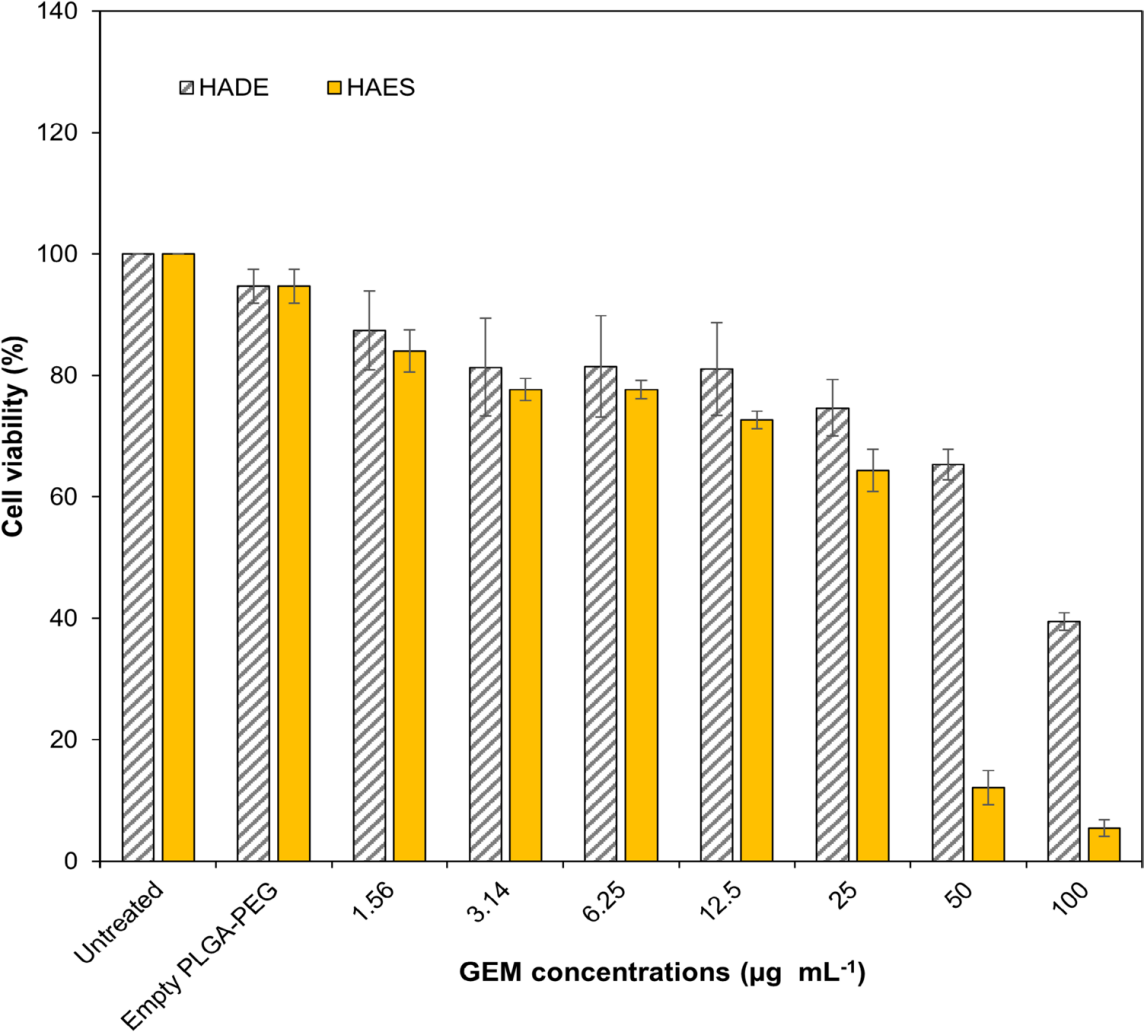
Cytotoxicity study of HAES and HADE. Cytotoxicity study was conducted using MTT assay at increasing concentration of 1.56 to 100 µg mL^−1^ using a HeLa cell line at 72 h incubation time. A spectrophotometer at 570 nm was used to determine the cell viability (%). HAES was shown to significantly have the lowest viable HeLa cells (%) at 50 and 100 µg mL^−1^ indicating higher toxicity compared to HADE. Results are expressed as mean ± SE (*n* = 3). Statistical analysis was performed using multiple t-tests: Significance is indicated as ***ρ < 0.00. Abbreviation: HADE-Hybrid AuNPs Double Emulsion, HAES-Hybrid AuNPs Electrospray

Figure 9 shows the MTT assay results of the HAES and HADE after 72 h of incubation, at different GEM concentrations ranging from 0 to 100 µg mL^−1^. The cytotoxicity effects of the HAES and HADE have increased as the concentrations of GEM increase indicating the dose-dependent anti-proliferation effects trend. HADE increased the cell proliferation inhibition (%) from 13 to 61% and HAES increased from 16 to 94.5% as the concentration increased from 1.56 to 100 µg mL^−1^. At 50 µg mL^−1^, HAES exhibited significantly higher cytotoxicity (~ 80% mortality) than the HADE (35% mortality). Similarly, at 100 µg mL^−1^, HAES showed almost 100% mortality (94.6%) and HADE only reached half of the mortality (~ 61.6%) indicating HAES exerted higher toxicity to HeLa cells in 72 h than HADE. Based on DL%, the nanoparticle mass was calculated, and showed a 21–32.5-fold increase in nanoparticles equivalent to the empty PLGA-PEG (control). However, this increase does not affect cancer cells, indicating that the observed toxicity was solely attributed to the GEM. The differences in HAES and HADE resulted in different physicochemical characteristics and drug release patterns. A 50% difference in cytotoxicity exerted by HAES on HeLa cells might be attributed to the size difference of HAES which is 43.8% smaller in size (147.00 ± 10.4 nm) than HADE, 287.47 ± 1.87 nm. Smaller particles help in cellular uptake resulting in higher bioavailability of the nanoparticles in the target cells thus inducing more mortality in the cells [74]. Furthermore, Fig. 5 and Fig. 7, showed that HAES have 23.61% faster and 13% higher release than HADE.

## Conclusions

Hybrid AuNPs have been successfully synthesised *via* two different techniques; electrospray and double emulsion. Electrospray synthesised smaller particle sizes (147.00 ± 10.4 nm) with long-term stability formulation (−31.5 ± 2.6 mV) that is suitable for intravenous delivery of GEM for tumour therapy compared to double emulsion (287.47 ± 1.87 nm, −23.30 mV). Moreover, HAES showed a 23.61% faster release than HADE likely due to the difference in distinct particle nanostructure formation that has been deduced based on previous research to understand the release profile. In addition, findings from this study showed a no-burst released profile in HAES suggesting that particles electrosprayed at varying temperatures help alter the composition of polymers, preventing burst effects during drug release. This finding shows the importance of electrospray in synthesising temperature-sensitive particles that can enhance the future of drug delivery systems. A 50% difference in cytotoxicity exerted by HAES than HADE was due to different physicochemical characteristics and drug release patterns. This report demonstrated the advantages of the electrospray-based synthesis method compared to conventional methods such as the double emulsion method. Morphological analysis and cellular uptake study can be conducted to improve the understanding at the cellular level.

## Data Availability

The datasets generated and/or analysed during this study are accessible on reasonable request from the corresponding author.
